# Advances in tumor microenvironment and underlying molecular mechanisms of bladder cancer: a systematic review

**DOI:** 10.1007/s12672-024-00902-8

**Published:** 2024-04-11

**Authors:** Liu Tang, Haifei Xu, Tong Wu, Wenhao Wu, Yuhao Lu, Jijia Gu, Xiaoling Wang, Mei Zhou, Qiuyang Chen, Xuan Sun, Hongzhou Cai

**Affiliations:** 1 Department of Nursing, Jiangsu Cancer Hospital and The Affiliated Cancer Hospital of Nanjing Medical University and Jiangsu Institute of Cancer Research, Nanjing, Jiangsu China; 2 Department of Urology, Nantong Tumor Hospital and Tumor Hospital Affiliated to Nantong University, Nantong, China; 3 Department of Radiology, Nanjing Medical University The Fourth School of Clinical Medicine, Nanjing, Jiangsu China; 4 Department of Urology, Jiangsu Cancer Hospital and The Affiliated Cancer Hospital of Nanjing Medical University and Jiangsu Institute of Cancer Research, Nanjing, Jiangsu China

**Keywords:** Molecular mechanisms, Tumor microenvironment, Bladder cancer

## Abstract

Bladder cancer is one of the most frequent malignant tumors of the urinary system. The prevalence of bladder cancer among men and women is roughly 5:2, and both its incidence and death have been rising steadily over the past few years. At the moment, metastasis and recurrence of advanced bladder cancer—which are believed to be connected to the malfunction of multigene and multilevel cell signaling network—remain the leading causes of bladder cancer-related death. The therapeutic treatment of bladder cancer will be greatly aided by the elucidation of these mechanisms. New concepts for the treatment of bladder cancer have been made possible by the advancement of research technologies and a number of new treatment options, including immunotherapy and targeted therapy. In this paper, we will extensively review the development of the tumor microenvironment and the possible molecular mechanisms of bladder cancer.

## Introduction

Bladder cancer (BC) is the tenth most common cancer worldwide with an estimated 573,000 new cases in 2020 [1]. The crude, age-standardized by China standard population and by world standard population rates were 5.80/10, 3.60/10 and 3.57/10for incidence, and 2.37/10, 1.31/10 and 1.32/10 for mortality, respectively [2]. The occurrence and progression of bladder cancer directly lead to life-threatening conditions for patients. Between males and females, the crude rate of bladder cancer is about 3:1. And the cumulative risk is much higher in males than in females [3]. Smoking is the most significant risk factor for BC [4], followed by work-related exposure to aromatic amines and polycyclic aromatic hydrocarbons. Environmental pollution, food, and genetic predisposition are other minor risk factors [5]. Bladder cancer can also be brought on by conditions including leukoplakia, urethral stones, and urinary retention. Due to the similarity of BC symptoms to those of a urinary tract infection, prompt diagnosis may be delayed. The majority of BC cases are only identified after a period of macroscopic hematuria [6].

BC is classified into two types: muscle-invasive bladder cancer (MIBC) and non-muscle-invasive bladder cancer (NMIBC). According to TNM staging, the degree of invasion and metastasis is the primary factor used to stage bladder cancer, while the primary factor used to grade bladder cancer is the characteristics of cell differentiation [7]. The TNM determines the original tumor (T), if lymph nodes are involved (N), and whether metastasis is evident (M). The grade of the disease determines how aggressively the disease progresses, with poorly differentiated bladder cancer being more likely to progress and spread than well-differentiated bladder cancer [8].

Tumor cells stimulate significant molecular, cellular and physical changes within their host tissues, resulting in a tumor microenvironment (TME). TME is a complex and evolving entity. The composition of the TME varies by tumor type, but hallmark features include immune cells, stromal cells, blood vessels, and extracellular matrix. In the early stages of tumor growth, a dynamic, reciprocal relationship develops between cancer cells and components of the TME to support cancer cell survival, local invasion, and metastatic spread. The characteristics of the TME include overall hypoxia, acidification, interstitial hypertension, vascular hyperpermeability, inflammatory reactivity, and immunosuppression. Tumors are also infiltrated by various adaptive and innate immune cells that can play both pro- and anti-tumorigenic roles [9]. In this essay, we will thoroughly examine the progress of the tumor microenvironment and putative molecular mechanisms of BC.

## Tumor immune microenvironment of BC

**Fig. 1:**
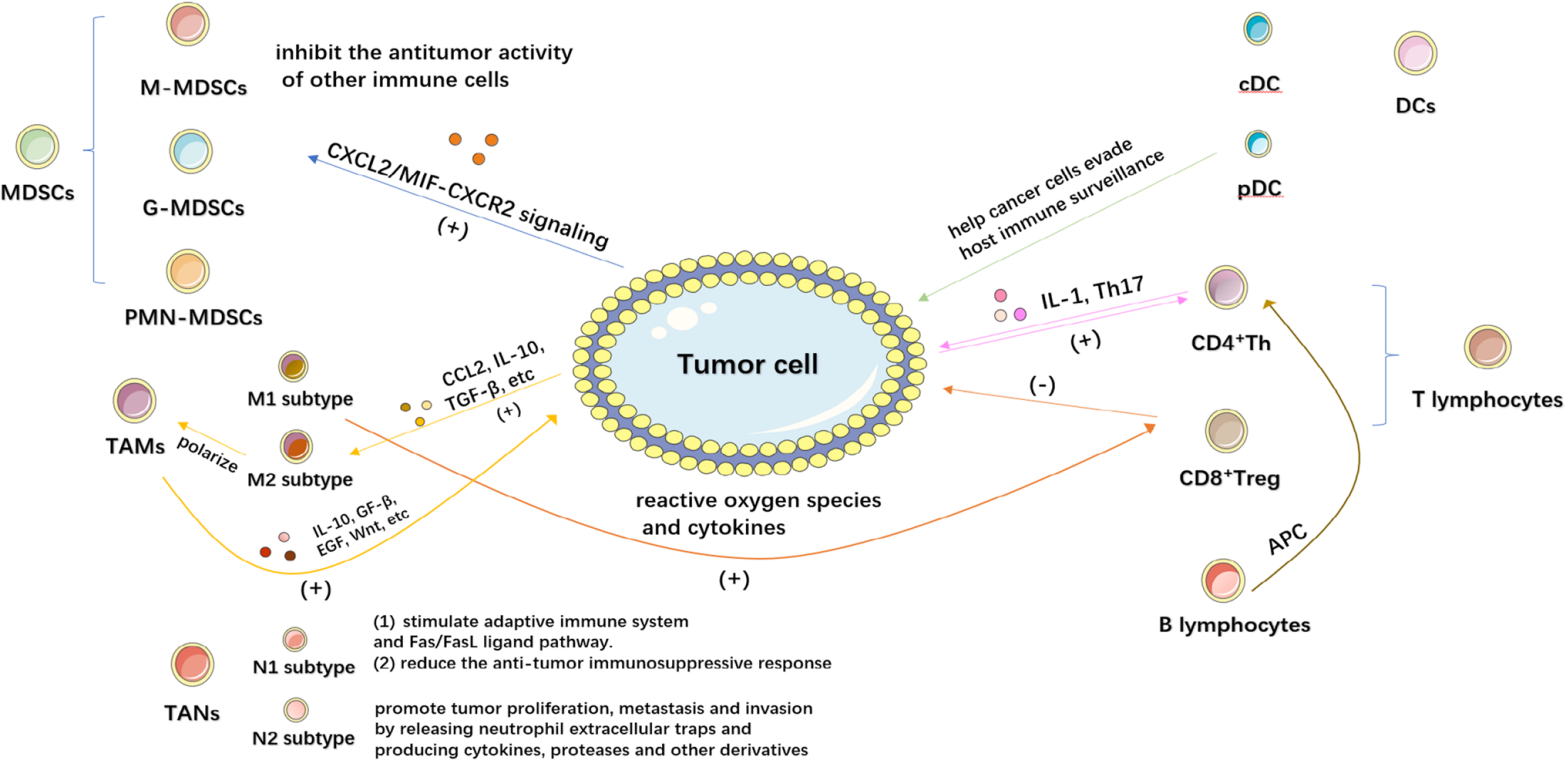
Tumor immune microenvironment of bladder cancer. This figure shows the composition of several major cell types in the bladder cancer tumor microenvironment and the roles of their subtypes. A set of arrows of the same color represents a complete action process, with a plus sign for promotion (or activation, recruitment) and a minus sign for inhibition (or elimination, killing)

The tumor immune microenvironment is a complex structure in which a large number of immune cells such as T cells, B cells, natural killer cells(NK cells), dendritic cells(DCs), tumor associate microphage cells(TAMs), tumor associate neutrophils(TANs) and myeloid-derived suppressor cells(MDSCs) exist. These cells interact with tumor cells and immunomodulatory molecules such as TGF-β, IDO, and Artemin within the TME [10]. In this section, we will provide a summary of the role and mechanism of the immune microenvironment in BC (Fig. 1).

### MDSCs in BC

MDSCs are a group of myeloid cells which interacts with tumor cells directly and inhibit the antitumor activity of other immune cells [11]. MDSCs can be classified into two phenotypes: monocyte MDSCs (M-MDSCs), which are similar to monocytes in appearance and phenotype, and granulocyte or polymorphonuclear MDSCs (G-MDSCs or PMN-MDSCs), which share phenotypic and physical characteristics with neutrophils. In healthy individuals, immature and progenitor bone marrow cells (IMCs) generated in the bone marrow rapidly differentiate into mature granulocytes, macrophages, or dendritic cells (DCs). However, in pathological situations such as cancer, numerous infectious diseases, sepsis, trauma, bone marrow transplantation, and several autoimmune diseases, the growth of this population—collectively referred to as MDSCs—is caused by the partial inhibition of IMC differentiation into mature bone marrow cells. [12].

In human BC, G-MDSC or total MDSC levels were higher in BC patients than in healthy, non-cancer controls. Human BC tissues predominately include G-MDSCs, and these cells are more prevalent in cancerous tissues than in nearby healthy tissues. Increased MDSCs are correlated with tumor size, pathological grade, and stage in peripheral blood or BC tissues. Group 2 innate lymphocytes (ILC2s), which in turn attract and activate MDSCs through the production of IL-13, are activated by BC cells through CXCL2/MIF-CXCR2 signaling and help to recruit MDSCs [13, 14]. Moreover, the MDSCs isolated from the peripheral blood of UBC patients could activate CD4^+^Foxp3^+^ Tregs cells and inhibit the T cell proliferative response [15].

In conclusion, Urothelial Bladder Cancer (UBC)is associated with an increased number of MDSCs in peripheral blood and tumor tissue. Tumor size, growth rate and subtype may correlate with the composition and number of MDSCs permeating peripheral blood and tumor tissues in UBC patients. Mechanistically, MDSCs secrete pro-inflammatory and immunosuppressive cytokines/chemokines that promote cancer-associated inflammation and immune evasion.

### TAMs in BC

Most tissues, including healthy bladders, contain macrophages, which are phagocytic immune cells. Their primary function is to phagocytose and digest cellular waste and pathogens while also stimulating other immune cells to fight off infections. Through their sentinel role, adaptability, and ability to respond to external physiological changes, they play a crucial role in preserving homeostasis [16]. TAMs are macrophages found in the TME and are frequently linked to the growth and metastasis of tumor cells. They can exhibit pro-tumor and anti-tumor capabilities in response to various polarizations [17]. To activate the body's defenses during inflammation, macrophages secrete inflammatory mediators. Despite the fact that these inflammatory macrophages are effective in the beginning, they can also cause significant tissue damage. As a result, macrophages enter an anti-inflammatory phenotype through apoptosis or polarization, reducing pro-inflammatory responses and speeds up wound healing [18]. Reactive oxygen species and cytokines activate cytotoxic T cells in tumor-associated macrophages of the M1 subtype, suppressing inflammation and inhibiting tumor growth [19]. In contrast, the M2 phenotype promotes bladder tumor development by activating cytotoxic T cells. According to research, early tumor cells can secrete CCL2, IL-10, TGF-β, etc. and other molecules to draw monocytes into the epithelium and polarize M2 macrophages into TAMs. TAM can then secrete IL-10, GF-β, EGF, WNT, etc. to influence the signal transduction pathway in tumor cells, leading to the proliferation and metastasis of tumor cells. As a result, TAM can encourage the occurrence and development of tumors by controlling relevant signaling pathways in tumor cells [20].

The occurrence, recurrence, and development of BC are all intimately associated with TAMs. In BC or tumors, post-polarized TAMs have a significant function. It has an impact on tumor growth, metastasis, infiltration, and even far-reaching metastasis. TAMs secrete a large number of cytokines (vascular endothelial growth factor, tumor necrosis factor-a, IL-8, TGF-β1, VEGF family, urokinase-type fibrinogen kinase, adrenomedullin and TNF-α). TGF-β1, one of these cytokines, encourages TAMs to secrete more VEGF by activating the T-RII/Smad3 signaling pathway [21]. In a study using in vitro cell culture, researchers discovered that by co-culturing TAMs with bladder cancer cell lines, they increased the levels of oncogenic markers like β-catenin and NF-κB and EMT markers like Snail, VEGF, and Vimentin, which were known to increase the metastatic potential of BC cells. More notably, silencing of miR-30 led in downregulation of Twist1 and Vimentin expression, lowering the metastatic potential of BC cells [22]. Co-culture also significantly boosted miRNA-30a expression in BC cells. Zhao et al. investigated the interactions between macrophages and cancer cells in the microenvironment of BC using a microfluidic coculture chip. It has been demonstrated that transitional cell carcinoma of the bladder (TCCB) cells polarize macrophages toward the M2 phenotype in a way that depends on the lactate flow between the cancer cell and the TAM [23]. They asserted that lactate shuttle is the primary cause of the immunosuppressive microenvironment in the TCCB and suggested that MCTs (monocarboxylic acid transporter) may be a fresh therapeutic target for TCCB [24].

From the numerous studies on tumor-associated macrophages (Table 1), it is evident that TAMs in the TME play a significant role in all stages of tumor immunosuppression, tumor growth, invasion, and metastasis, and that any of these processes may result in a significant advancement in the study of BC. Additionally, we think that our research on tumor-associated macrophages will soon help BC patients and give doctors additional choices for treating the disease.

**Table 1:** Molecular pathways and chemokines associated with the development of bladder cancer with TAMs

Factors	Findings	References
miR-30a/NF-κB/Snail	TAMs have been shown to produce different interleukins such as IL-10, vascular endothelial growth factor, and IL-8, which promote and mediate angiogenesis in the tumor microenvironment through NF-κB-mediated signal transduction, thereby promoting the development of bladder cancer	[22]
COX2/mPGES1/PGE2	COX2/mPGES1/PGE2 signaling regulates PD-L1 expression in tumor-infiltrating myeloid cells such as TAM and MDSC. The increased expression of PD-L1 in tumor-recruited myeloid cells is a mechanism by which tumors evade the immune system	[203]
RSPO/LGR4/ERK/STAT3	G protein-coupled receptor 4 promotes the M2-type polarization of monocyte-macrophages through the RSPO/LGR4/ERK/STAT3 signaling pathway	[204]
IL-10/STAT3	Macrophages exposed to hypoxic tumor core lysates in vitro exhibited high IL-10, HIF1α and VEGF expression that was significantly downregulated by NLGP. Neem leaf glycoprotein (NLGP) converts M2-type TAMs to M1-type TAMs. This TAM-regulatory function of NLGP may contribute by increasing the proportion of M1-type TAMs in the tumor core	[205]
α2β1/PI3K/AKT	Collagen secreted by TAMs can activate the PI3K/AKT signaling pathway through integrin α2β1 to promote bladder cancer proliferation	[206]
CXCL1	Chemotaxis of CXCL1 and IL-6 and hypoxic conditions induce and recruit TAMs and CAFs into tumor areas, leading to cancer cell aggregation that favors the tumor microenvironment. Both TAMs and CAFs provide CXCL1 and other growth factors as feedback to cancer cells, which promotes close communication and interaction between cancer cells and TAMs/CAFs	[207]
LNMAT1/CCL2	LNMAT1-induced upregulation of CCL2 recruits macrophages into the tumor, which promotes lymphatic metastasis via VEGF-C excretion	[208]
MCT	Polarization of macrophages toward M2 phenotype in a manner that depended on cancer cell-TAM lactate flux	[24]
BMP	BMP4 secretion by bladder cancer cells provide macrophage polarization toward M2 phenotype	[209]

### TANs in BC

The N1 and N2 subtypes of TANs can be polarized in response to various cues in TME. Neutrophil cytotoxicity, activation of the adaptive immune system, and activation of the Fas/Fas ligand pathway are the key ways that N1 neutrophils promote cytotoxicity. To achieve the goal of tumor suppression, they might also reduce the anti-tumor immune response. In contrast, N2 neutrophils promote tumor growth, metastasis, and invasion primarily by releasing neutrophil extracellular traps and generating cytokines, proteases, and other derivatives [25]. The two phenotypes of TANs can transition into one another, despite the fact that the mechanism underlying TANs' polarization is still unknown. And no unique marker has been discovered to distinguish between N1 and N2 neutrophils till now. When the Transforming growth factor -β (TGF-β) was present or the Colony stimulating factor (G-CSF) was stimulated, the infiltration of N2 neutrophils in TME gradually increased. When Interferon type I (IFN) or TGF-β is blocked, TANs in mice and humans can be transformed into anti-tumor type N1.

The following two factors now influence neutrophils' participation in the treatment of BC: Before surgery, the neutrophil–lymphocyte ratio (NLR) can be examined to determine the prognosis of BC. The other significant risk factor influencing the prognosis of BC is the expression of HNP-1, HNP-2, and HNP-3.

According to MANO et al.'s study of the recurrence and progression of 107 patients after transurethral resection of bladder tumors, NLR > 2.41 was associated with tumor progression, while NLR > 2.43 was associated with tumor recurrence [26]. They discovered NLR as an independent predictor of disease progression and recurrence in patients with bladder cancer that is not muscle-invasive. NLR was associated with tumor recurrence but not tumor progression, according to a different international study on the recurrence and progression of 166 patients with high-grade T1 BC [27]. This viewpoint is also supported by a single center study by FAVILLA et al. [28] The study of NLR and tumor recurrence and progression should take age into account, according to ALBAYRAK et al. [29]. As a result, bigger sample size future randomized controlled studies are required to demonstrate the relationship between NLR and tumor progression and recurrence in patients with non-muscle invasive bladder cancer.

According to studies, the primary source of eosinophil and neutrophil invasion in tumors is the expression of HNP1-3 [30]. However, studies have revealed that the extra HNP1-3 found in the urine of BC patients is actually created by the cancer cells in the bladder, and that highly invasive bladder cancer cells produce more HNP1-3 than less invasive bladder cancer cells [31]. The upregulation of HNP1-3 in tumors may arise mostly from invading immune cells but may also be started by HNP1-3 generating cancer cells because it is unknown whether the epithelial expression of HNP1-3 is increased by an inflammatory state or is released by nearby neutrophils and picked up by epithelial cells [32].

Table 2 provides more details about the role of TANs in BC.

**Table 2:** The role of TANs or their protein concentrations in bladder cancer

Concentration of neutrophils or their proteins	Effect	References
HNP-1	Evaluating prognosis	[210]
HNP-2	Evaluating prognosis	
HNP-3	Evaluating prognosis	
NLR	Evaluating prognosis	[211]
ARG-1	Promote tumor metastasis	[212]
IL-2	Inhibit the growth of tumor	[213]
IL-4	Inhibit the growth of tumor	
G-CSF, IL-6	Promote tumor angiogenesis	[214]
CCL-2	Proinflammatory chemokines	[15]
CCL-3	Proinflammatory chemokines	
CCL-4	Proinflammatory chemokines	

### DCs in BC

The development of innate and adaptive immunity begins with DCs, which are also the best antigen-presenting cells for triggering antigen-specific antitumor immunity [33]. Human circulating DCs can be broadly categorized into two groups: CD11c ^neg^ CD123^+^ plasmacytoid DCs (pDCs) and conventional CD11c^+^ CD123^neg^ DCs (cDCs) [34]. Differentiated cDCs are initially immature and require maturation cues (such as damage or pathogen-associated molecular patterns [DAMPs or PAMPs] or inflammatory cytokines) to completely perform their function in the immune response [35]. Mature DCs are capable of priming naive T cells and starting adaptive immune responses because of phenotypic changes that take place during activation. Depending on the conditions of activation, dendritic cells can exhibit either inflammatory or tolerant phenotypes. In the TME, dendritic cells frequently have a repressed and defective phenotype, which aids cancer cells in evading host immune surveillance [36].

DCs contribute significantly to tumorigenesis in BC. Troy et al. reported that tumor-infiltrating DCs in BC tissue were predominantly immature and significantly reduced in number compared to DCs in normal bladder tissue. The low infiltration and functional defects of DCs resulted in non-effective antigen presentation and the expression of costimulatory and adhesion molecules was too low to induce specific CTLs responses, ultimately leading to the immune escape of BC [37]. Additionally, CXCL9 released by dendritic cells associated with tumors increased the expression of PD-L1 in BC cells through stimulating CXCR3 signaling [38]. A cancer cell's upregulated PD-L1 expression should limit the activation and operation of antigen-presenting cells and effector T cells because PD-L1 is a potent inhibitor of antitumor T cell immunity. This will help the cancer cell avoid being targeted by antitumor responses.

Given that DCs play a significant role in antitumor immunity and that a functional deficit in them is one of the primary factors in the development of urologic tumor it is practically feasible to increase the quantity and activity of mature DCs within the tumor by using DC vaccinations or other techniques in order to efficiently transmit tumor antigens and strengthen the immune system's capacity to destroy tumors. For BC immunotherapy, this would be a fresh starting point.

### CD4 T lymphocytes in BC

The T helper cell (Th), which includes the Th1, Th2, Th17 subpopulations and Treg, is produced by CD4-positive T cells. Th, found in TME, has been associated to a longer period of BC patient survival without recurrence [39].

The roles of Th17 cells in autoimmune disorders and inflammation have been extensively studied. The autocrine action of the IL-21 that they secrete increases Th17 cell production [40]. It also promotes the expression of the IL-23 receptor on those cells, making Th17 cells receptive to IL-23 activation [41].

Treg cells that express the FOXP3 transcription factor play essential roles in immune homeostasis and self-tolerance, which are immunosuppressive and reduce effector T cell proliferation to promote tumor survival [42]. Treg cells have the ability to reduce the effectiveness of antitumor immunity through a number of immunosuppressive mechanisms, both in a contact-dependent way and by secreting inhibitory cytokines; these effects cumulatively lead to tumor progression [43]. Treg has consequently become a highly important therapeutic target in the treatment of cancer and also has the potential to improve the efficacy of cancer and infectious disease vaccines. Treg is frequently elevated during cancer and chronic infections as a mechanism of immune subversion [44].

BC is more capable of drawing T cells than the normal bladder tissues in its immediate vicinity. Additionally, enhanced BC metastasis was one of the effects of the greater levels of recruitment. Th cells may work by secreting the cytokine IL-1, which boosts the BC androgen receptor (AR) signaling, promotes the recruitment of T cells there, and accelerates BC cell invasion by up-regulating the expression of HIF-1α/VEGFα. Interruption of the IL-1 → AR → HIF-1α → VEGFα signals with inhibitors of HIF-1α or VEGFα partially reversed the enhanced-BC cell invasion [45]. ELISA study showed that the circulating levels of IL-17, IL-23, and IL-6 (cytokines related to Th17) were greater in bladder cancer patients, which may indicate a significant function for these cytokines in the differentiation and proliferation of Th17 cells in this illness. With Th17 cells as the target, a more effective therapeutic approach should be developed. Additionally, previous studies have found a beneficial relationship between Treg infiltration and survival in urinary BC [46]. Studies have discovered an accumulation of CD4 + FOXP3 + T cells, which are Treg cells, in the BC. In vitro MMP2 expression is controlled by Tregs, which also prevent the formation of the invasion-promoting factor MMP2 in the tumor microenvironment [47]. Tregs might therefore encourage BC metastasis.

### CD8 T lymphocytes in BC

Bone marrow hematopoietic stem cells are the source of CD8 + T cell development. They grow and mature in the thymus before migrating outside of it to move between the lymphoid and blood organs [48]. It is separated into two categories: killing CD8 + T cells and reviving worn-out CD8 + T cells. More and more data points to the possibility that reduced CD8 + T cells may experience metabolic insufficiency, along with modifications to signaling cascades and the epigenetic backdrop that depress effector immunity and impair response to immune checkpoint blockade therapy. Early depletion CD8 + T had different metabolic modifications from effector and memory T. These differences were mostly due to the suppression of aerobic glycolysis and mitochondrial respiration, which was shown as a restriction on glucose uptake and utilization, mitochondrial depolarization, and excessive ROS production [49].

CD8 + T cells can not only play a protective role in the treatment of BC, but they can also act as a sign of a favorable prognosis. They can take part in both the direct reaction between cells, which they can do by releasing granase, and the favorable immune response, which they can do by metabolizing cytokines like IL-2 and IFN-γ to suppress tumors, as described in the referenced study [50]. For the purpose of eliminating tumor cells, regulatory CD8 + NKT cells are crucial. Effector cells directly cause the death of tumor cells in this way. According to a study by Lu Ting et al., the expression of CD4 + T lymphocytes in the tumor tissues of MIBC patients was significantly higher than that in nearby tissues, whereas the expression of CD8 + T lymphocytes was significantly lower than that in neighboring tissues.

### B cells in BC

The majority of B cells are thought to form soluble immune cells. Immunoglobulins of various subclasses are utilized depending on the pathogen binding requirements. The B cell receptor, which is carried by memory membrane-bound immunoglobulins on B cells, mediates the uptake of circulating antigens with the intention of internalizing, degrading, and presenting them as antigenic peptides in the MHC class II pocket of CD4 + Thelper cells. Successful B cell activation causes clonal proliferation and class switching, resulting in the maturation of antigen-specific antibodies and the generation of memory B cells [51].

B cells also have a significant impact on TME. Similar to T cells, BC tissue also attracted more B cells than the usual BC tissue around it, and these attracted B cells likewise enhanced BC cells' capacity for invasion. The specific method may involve the ability of B cells to increase IL-8/AR signaling in BC cells, which would then promote the expression of metastatic genes such as matrix metalloproteinase 1 (MMP1) and MMP13 [52]. It is still necessary to investigate a more specific mechanistic investigation of B cells and BC metastasis. Future clinical studies demonstrating the use of anti-IL-8 neutralizing antibodies, AR-siRNA, or MMPs inhibitors to inhibit IL-8/AR/MMPs signaling to partially reverse the ability of B cells to invade BCA for the treatment of BC and to prevent the further development of BC point to new research directions [53].

### NK cells in BC

NK cells play a crucial role in the immune response due to their ability to express a variety of receptors that can identify both cells transformed by pathogens and cells infected by them [54]. NK cells are the primary effector cells in innate immunity against cancer and exhibit high heterogeneity in the tumor microenvironment. While most current therapeutic regimens targeting the tumor microenvironment focus on T-cell immunity, either by promoting activating signals or inhibiting suppressive signals. NK cells immunotherapy has now yielded some results. Although tumors also evolve to resist NK cell-induced cytotoxicity, strategies such as cytokine support, blockade of inhibitory molecules, and genetic engineering of NK cells may overcome this resistance and hold great potential for treating both solid tumors and hematologic malignancies [55].

The researches have shown that tumor cells are more vulnerable to NK cell destruction when inhibitory receptor ligands, such MHC-I, are downregulated in combination with the abundant expression of ligands recognized by activating receptors, like the killer cell lectin-like receptor K1 (KLRK1) and the natural cytotoxicity receptors (NCRs) [56–58]. NKG2D, also referred to as human KLRK1, is a stress-inducible ligand that is expressed on malignant cells and is recognized by NK cells and CD8 + T lymphocytes. Examples of these stress-inducible ligands include MICA, MICB, and the ULBP-binding proteins 1–6, which are collectively expressed to as "NKG2D ligands" (NKG2D-L) [59, 60]. In vitro tests do in fact show that one of the main ways that BC tumor cells are recognized is through NKG2D binding to stress-inducible ligands [61]. Numerous other activating and inhibitory receptors that are expressed by NK cells are anticipated to work together to promote the highest levels of NK cell activity in the tumor microenvironment [62]. Similar to NKG2D, TNFRSF14 expresses on NK and CD8 + T lymphocytes and possesses a number of ligands, including TNFSF14 (also known as LIGHT). Depending on the cis and trans contacts with the ligand, TNFSF14 can transmit either lymphocyte activation or inhibition. For instance, functioning as a receptor for TNFSF14 or LTA, TNFRSF14 can activate downstream NF-kB signaling to boost NK cell and T-lymphocyte proliferation, IFN-g production, and tumor cell clearance [63–67]. Using CIBERSORT, Yuhan Sun et al. produced transcriptional signatures for NK cell phenotypes that were at rest, enlarged by IL-2, and triggered by PDGF-DD, and determined how common each was in the bladder cancer (BC) dataset from The Cancer Genome Atlas [68]. First, studies show that NK cells that have been exposed to IL-2 are associated to a better prognosis for BC. The IL2NK phenotype is more prevalent in low and high clinical grades of BC tumors, followed by the SPANK and then the ReNK phenotypes. Additionally, different clinical grades of BC tumors exhibit an abundance of NK Cell Phenotypes [56]. Table 3 provides more specifics regarding how NK cells work in BC.

**Table 3:** Molecular pathways and chemokines associated with the development of bladder cancer with NK cells

Serial number	Related research findings	Key words	References
1	NK cells highly express CD28H with naïve and poorly differentiated properties, and repeated antigen stimulation leads to permanent loss of CD28H B7-H5 is a ligand for CD28H and is widely expressed in tumor cells which relates to a worse prognosis for bladder urothelial carcinoma (BUC)	CD28H, B7-H5, BUC	[162]
2	The IL-2-expanded NK cell phenotype was the most abundant in low and high grades of the Cancer Genome Atlas bladder cancer (BLCA) tumors and was associated with improved prognosis	IL-2, BLCA	[56]
3	Platelet-derived growth factor D (PDGF-DD) expression was associated with numerous cancer hallmark pathways in BLCA tumors compared with normal bladder tissue, and a high tumor abundance of PDGFD transcripts and the PDGF-DD-activated NK cell phenotype were associated with a poor BLCA prognosis	PDGF-DD, BLCA	[56]
4	High tumor expression of transcripts encoding the activating NK cell receptors-KLRK1 and the CD160–TNFRSF14 receptor–ligand pair, was strongly correlated with the IL-2-expanded NK cell phenotype and improved BLCA prognosis	KLRK1, BLCA prognosis	[58]
5	IL-2c enhances NK cell activation and maturation in orthotopic BC. NK cells are indispensable for efficacious treatment of lung metastatic BC	IL-2c, lung metastatic BC	[215]
6	A high level of IL-10^+^TAMs is associated with a state of high immune infiltration, with multiple immune cells enriched in the TME. but immune effector cells (CD8 + T cells and NK cells) present an exhausted or dysfunctional state, and therefore, could not play an active role in anti-tumor immunity	IL-10^+^TAMS, immune infiltration, anti-tumor immunity	[216]
7	The contribution of NK cells in the elimination of human bladder cancer has been extensively reported with the tuberculosis vaccine Bacille Calmette-Guérin (BCG), which is the treatment of choice for T1G3 non-muscle invasive bladder cancer (NMIBC) There is a strong Natural Killer (NK) cell component in the mechanism of action of BCG in bladder cancer evidenced by the proliferation and activation of BCG-stimulated CD56^bright^ NK cells, hereafter, referred to as CD56^bright^ NK cells	BCG, NMIBC, CD56^bright^	[54, 217]
8	Natural killer cells had been suggested to play a role in the recognition of J82 and T24 bladder tumor cell lines. The main receptor involved in the recognition of bladder cancer cells by NK cells is NKG2D, which is present in all NK cells. However, in some cases, modulation of the response by NKp46 and adhesion molecules can contribute to immune recognition by NK cells in this system. NKG2D-specific mAb blocked recognition of bladder cancer cells both in degranulation and cytotoxicity experiments,	J82, T24, NKG2D, NKp46	[61, 217]

## Major stromal cells in the tumor microenvironment of BC

**Fig. 2:**
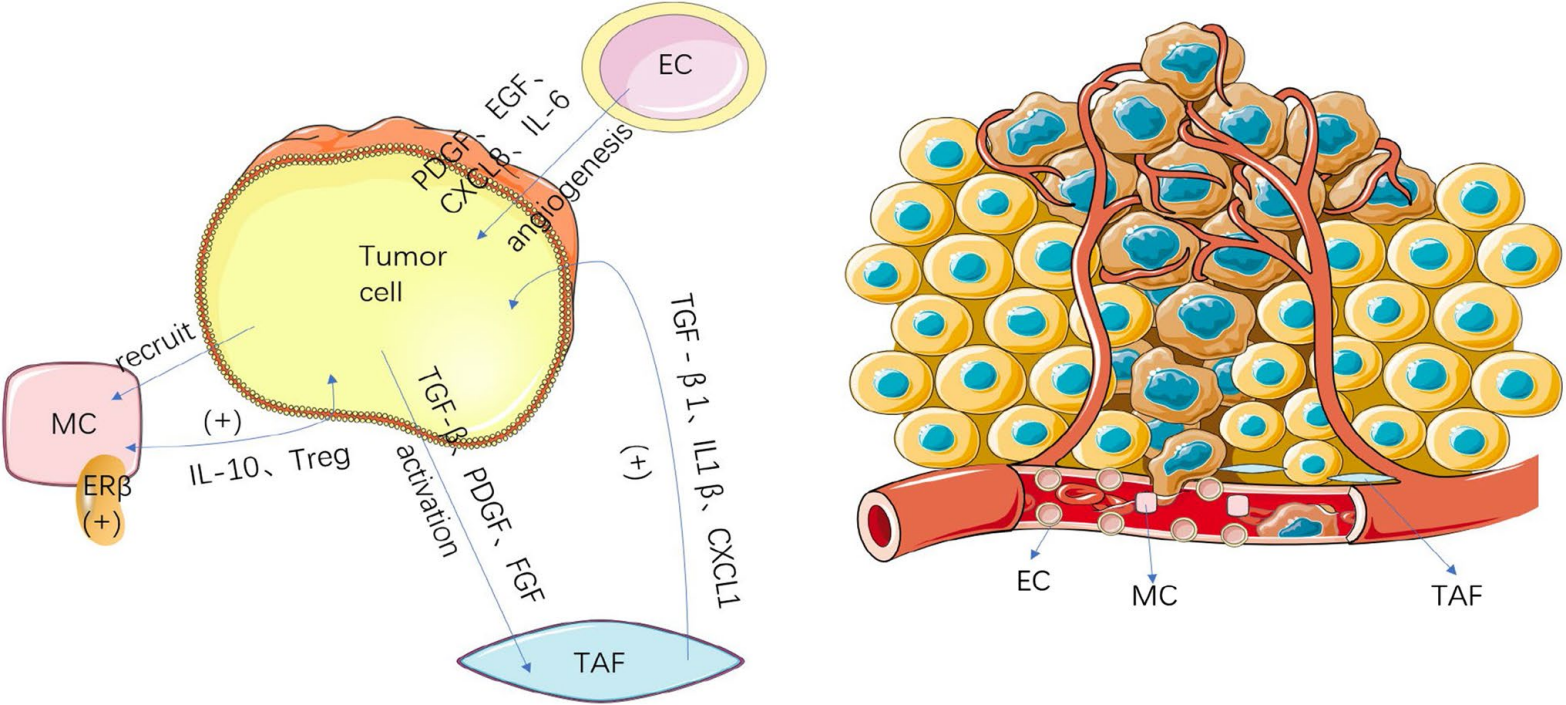
Major stromal cells in the tumor microenvironment of bladder cancer. This figure shows the composition of several major stromal cells in the bladder cancer tumor microenvironment and their associated mechanisms that promote the development of bladder cancer

To facilitate development of tumors, cancer cells recruit supportive stromal cells from the stroma of neighboring endogenous tissues, making stromal cells a crucial part of the TME. Vascular endothelial cells (ECs), tumor associate fibroblasts(TAFs), adipocytes, and mast cells (MCs) are among the stromal cells, which vary widely in composition depending on the kind of tumor. Upon recruitment to the TME, stromal cells secrete a variety of substances that have an impact on BC angiogenesis, proliferation, invasion, and metastasis [69] (Fig. 2).

### TAFs in BC

TAFs are a significant component. The activation of TAFs is dependent on tumor cells, and TAFs themselves contribute the malignancy of tumor cells. TAFs are primarily found surrounding tumor vascular endothelial cells or at the front of the tumor invasion. When it is exposed to tumor-related active mediators like transforming growth factor β (TGF-β), platelet derived growth factor (PDGF), and fibroblast growth factor (FGF), which are normally present in a dormant state, it enters an activated state and differentiates into a class of fibroblasts with properties similar to myofibroblasts. They do this by secreting a variety of cytokines, which control the progression and metastasis of tumor cells [70].

Research on the role of TAFs in BC is still in its early stages. Zhang et al. [71] demonstrated that TAFs in the tumor microenvironment up-regulate TGF-β 1, which can increase the ability of tumor cells to undergo epithelial stromal transition and encourage BC metastasis. Yang et al. showed that TAFs can release IL-1β to increase the invasion and proliferation of BC T24 cells. Miyake et al. [72] found that cancer-associated fibroblasts (CAFs) with high CXCL1 expression can significantly increase the invasion and migration of BC cells and may also indicate a poor prognosis. TAFs in the TME influence BC progression in various ways. A novel approach to BC treatment may involve identifying and targeting these mechanisms.

### MCs in BC

MCs are located throughout the visceral mucosa and the microvessels beneath the skin. It contributes to the control of the immune system and secretes a variety of cytokines. MCs are non-circulating immune cells that do not mature until they have reached the tissue they are intended to colonize. The final MC phenotype is greatly reliant on the microenvironment in which they are found after these tissue MCs undergo a number of stages of maturation that are predominantly mediated by the c-kit ligand, stem cell factor.

The recruitment of MCs has been associated to an improvement in BC. The recruitment may facilitate the metastasis of BC. MCs are present in the mucosa of bladder, which allows them to come into contact with the outside environment. Although MCs can actively destroy tumor cells [73], they can also promote tumor growth through a sophisticated mechanism. The upregulation of estrogen receptor beta (ERβ) in MCs has been identified as a potential contributing factor.

Research findings suggest that BC tissues may attract more MCs than normal tissues, increasing BC cell invasion. Co-culturing with mast cells has been shown to increase estrogen receptor beta mRNA expression, which is one of the genes associated with invasion progression. This evidence supports the notion that inhibiting ERβ in BC cells could prevent invasion. In summary, infiltrating mast cells can activate ERβ to promote BC cell invasion. Similarly, ERβ-CCL2 signaling can be enhanced by recruited MCs. Additionally, CCL2 is associated with EMT signaling pathways and can increase MMP9 signaling to further facilitate invasion, contributing to BC metastasis [74]. Interestingly, MCs do not always aid in invasion. By recruiting CD8 + T cells, MCs can actively inhibit early-stage tumor development [75]. The ability of the MCs to promote tumor growth by upregulating IL-10 expression does not become available until the late stages. Additionally, MCs can also move Treg cells. The effect of MCs on tumor growth is expected to be reversed by endogenous DAMP molecules.

### ECs in BC

Tumor angiogenesis is an important feature of the tumor microenvironment and plays a crucial role in bladder cancer progression. These complex processes involve the formation of new blood vessels in response to interactions between bladder cancer cells and endothelial cells (ECs). It has been shown that bladder cancer cells and vascular ECs secrete growth factors that enhance the proliferation and migration of both cell types through their interactions [76]. Since tumor vessels supply most of the oxygen and other essential nutrients to tumor tissues, angiogenesis inhibitors that target vascular endothelial growth factor (VEGF) and myeloid cells in tumor vessel vascularization are used to block tumor blood supply. Therefore, blocking the supply of endothelial cells, particularly the energy supply for glycolysis, may be beneficial for antiangiogenic therapy [77].

The study demonstrated that the VEGFR-2 signaling pathway was activated in EC-secreted EGFR ligands via the interaction between bladder cancer cells and ECs; furthermore, vascular EC-secreted epidermal growth factor receptor (EGFR) ligands binding to their receptors on bladder cancer cells induced proliferation, migration and invasion through EGFR signaling and induced the secretion of CXC chemokines from bladder cancer cells, to enhance ECs recruitment. Anyway, ECs have an active role in bladder cancer. EGFR was phosphorylated in bladder cancer cells by the EC-secreted EGFR ligands and EGFR ligands, including epidermal growth factor (EGF), amphiregulin, epiregulin, betacellulin, tumor necrosis factor-α and epithelial mitogen are upregulated in EC by co-culturing [78].

## Metabolomic analysis in BC

In order to better understand its pathological behavior, BC has also been studied by metabolomics method. Metabolites, which are the end-products or downstream, intermediate, low-molecular-weight products of metabolic pathways (e.g., glucose, lipids, amino acids, nucleotide metabolites), have been implicated in different functions in cancer progression, such as immune escape, differentiation, apoptosis, and cell invasion.

Metabolomics is defined as the study of metabolites in a biological sample (e.g., urine, blood) involved in the regulation of catabolic and anabolic pathways under specific physiological or pathological conditions [79]. Metabolomic analysis is powerful family of tools mainly often used for study of biofluids. Small molecules levels in biofluids reflects the current state of the organism allowing for identification and characterization of potential disease biomarkers. The number of metabolomics studies in the diagnosis and understanding of many diseases is rapidly growing in recent years [80].

A metabolomics approach has been implemented to study the tumorigenesis of different cancers, focusing on pathogenesis and biomarker research. This approach has contributed to our understanding of the relevant alterations in catabolic and anabolic processes that are impaired in cancer cells. Over the past fifteen years, metabolomic analytical methods also have been used extensively to investigate BC and to identify potential biomarkers of this cancer in urine, serum, and tissues [81]. Compared to urine, serum metabolomics is less prone to be affected by dilution factor. Serum is also more readily available than tissue and procedure less invasive [82]. Despite the advantages of examining the metabolomes of human sera, there are only a few studies on serum metabolomics focused on BC biomarker discovery. So far, most studies related to the analysis of serum of patients with bladder cancer have been carried out using NMR or mass spectrometry coupled with liquid and gas chromatography (GC) [83].

BC metabolomic profiling is usually the first step in the definition of specific cancer related pathways. Thanks to the integration of metabolomic and transcriptomic information, altered metabolites and lipids have been linked to their corresponding genes. An in-depth pathway analysis has been performed to discover how metabolic pathways (glucose, lipid, amino acid, and nucleotide metabolites) are perturbed in different grades of bladder cancer.

Metabolism is considered one of the key characteristics of cancer. Cancer cells tend to use glycolysis as an alternative to the aerobic cycle (oxidative phosphorylation) of normal cells, and therefore use the mitochondria differently, which is known as the Warburg effect [84]. Therefore, clarification of the mitochondrial processes and mechanisms for regulating the aerobic cycle and glycolysis have been an important focus in the research of bladder tumors. Many metabolites and genes involved in the Warburg effect have been detected in BC metabolomic studies. Petrella et al. [85] observed that alanine excretion was higher for RT4 cells, showing a very clear trend between different grades of malignancy. The pyruvate produced by glycolysis can either be transformed into lactate in the cytosol or enter the mitochondria, where it can be converted to alanine through the transamination reaction. The value of lactate excretion is directly proportional to the degree of glycolysis activity, whereas the degree of alanine excretion can be used as a measure of mitochondrial and oxidative phosphorylation (OxPhos) activities. For this reason, the lactate/alanine ratio is a metabolic measurement of the aerobic/anaerobic balance.

The lipid metabolism also plays a key role in cell motility, cell invasion, and tumor metastasis. It has been suggested that a perturbation in phospholipid metabolism is associated with tumor progression and aggressiveness. Differences in TGs levels between HG and LG BC lines may reflect the distinct reliability in β-oxidation to generate energy. Decreased levels of myristic, palmitic, and palmitoleic acids have been described in high-grade cell lines. These findings are consistent with the need to obtain energy through FA β-oxidation to continue growing and proliferating [86].

Metabolomics is a promising field for the discovery of BC biomarkers. However, some challenges and limitations remain, as most studies are limited to small populations and have not been validated by quantitative standardized methods. Metabolite analysis revealed aberrant metabolic pathways in BC, and these findings suggest its potential function as a biomarker for early detection of BC [87].

## Microenvironment promoting angiogenesis in BC

**Fig. 3:**
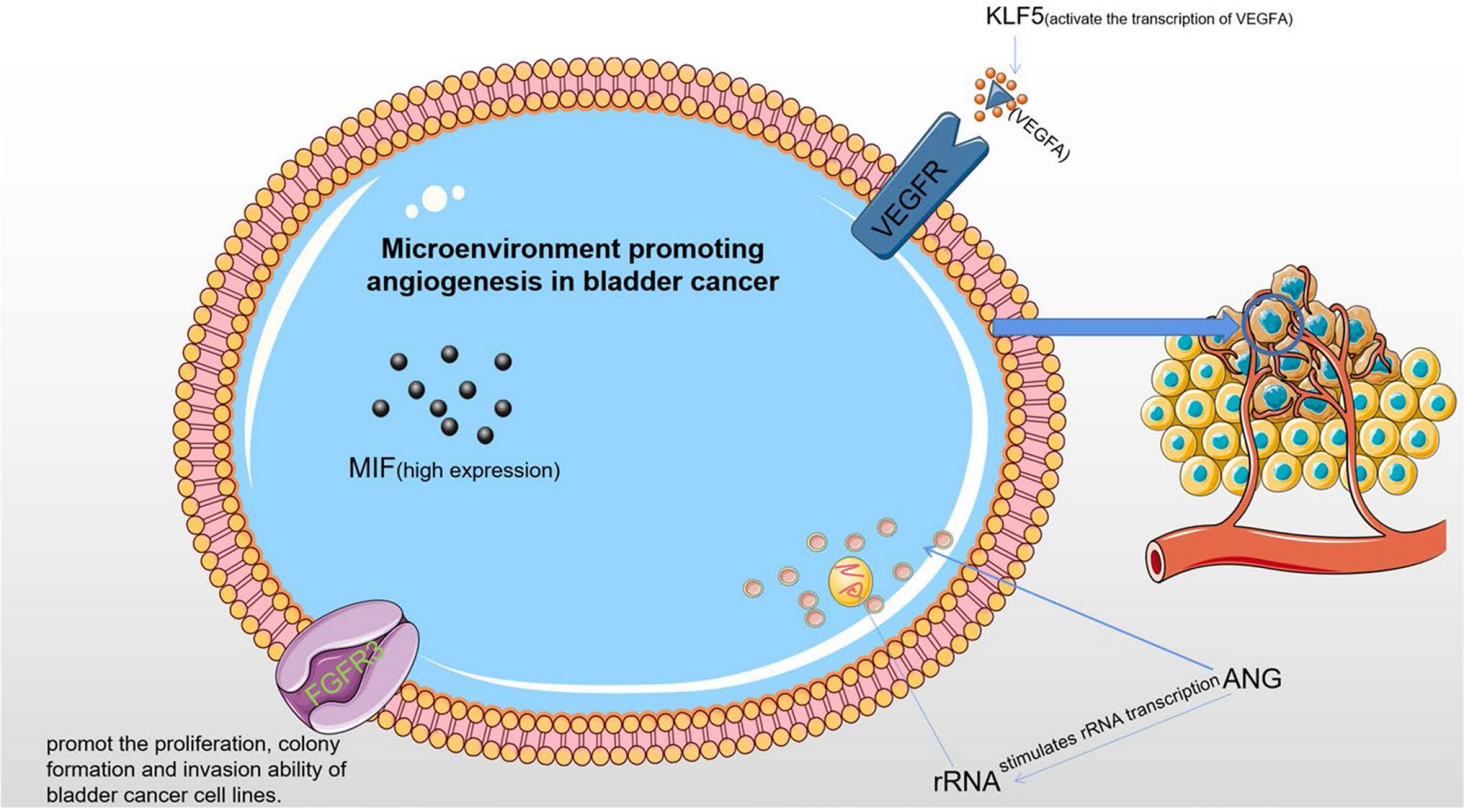
Microenvironment promoting angiogenesis in bladder cancer. In tumor vascular endothelial cells, ANG stimulates rRNA transcription to promote tumor angiogenesis. KLF5 directly regulates the transcription of VEGFA to promote bladder cancer angiogenesis. MIF promotes the invasion and metastasis of bladder cancer cells

Angiogenesis is a significant component of the tumor microenvironment and is thought to play a major role in the progression and metastasis of BC (Fig. 3). The primary mechanism of tumor pro-angiogenesis is the secretion of different cytokines by tumor cells and mesenchymal cells, which stimulate tumor microangiogenesis and control tumor cell proliferation, whereas corresponding inhibitors can prevent tumor growth.

### Angiogenin (ANG)

ANG, a multifunctional protein nd member of the pancreatic ribonuclease superfamily, is essential for the development and growth of tumor blood vessels. It is now known that ANG activates the putative receptor on endothelial cells, which in turn activates the cell signaling pathway. Cell survival, growth, proliferation, migration, tube formation, and tumor angiogenesis are just a few of the biological processes that ANG is linked to [88]. Additionally, it is linked to the development of tumors and a poor prognosis for patients [89].

ANG-induced angiogenesis and other biological processes may depend on the interaction of the ANG and AKT signaling pathways, which is associated with cell growth, apoptosis, and cell-cycle progression. AKT has been shown to be activated by ANG and can cause prostatic intraepithelial neoplasia (PIN) in mice [90]. The AKT/mTOR pathway mediates the synthesis of ribosomal proteins and crosstalk may coordinate a carefully planned synthesis of ribosomes. The AKT/mTOR signaling pathway, which is often activated in a variety of cancers [91], is regarded to be a key mediator in signal transduction pathways. Several studies have reported increased ANG levels in the urine of BC patients. Research has shown that downregulating ANG can regulate cell shape, control cell cycle, inhibit cell proliferation, and trigger cell death. Both cancer cells and endothelial cells have demonstrated nuclear translocation of ANG, which activates rRNA transcription. An animal investigation demonstrated that down-regulating ANG might significantly inhibit tumor xenograft growth, tumor angiogenesis, and tumor metastasis. In conclusion, ANG might be a useful target for both diagnostic and therapeutic interventions to manage BC [92].

### Human Kruppel-like factor 5 (KLF5)

The human Kruppel-like factor 5 (KLF5/IKLF/BTEB2), the fifth member of the Kruppel-like family, has demonstrated significant roles in various physiological and pathological processes, including embryonic development, cellular proliferation and differentiation, stress response, cardiovascular remodeling, and carcinogenesis by regulating the transcription of its target genes [93].

Degradation of KLF5 protein can inhibit tumor growth in breast cancer (BC) cells [94]. KLF5 is also crucial for the establishment and final urothelial differentiation during bladder development in mice [95]. Recent studies have suggested the relevance of the KLF5 gene in BC by finding mutations in up to 8% of muscle-invasive bladder cancer (MIBC). A study using lentivirus-mediated KLF5 knockdown showed that KLF5 not only revealed its pro-proliferative role in BC cell lines but also regulated the interaction between BC cells and vascular endothelial cells in vitro and promoted BC angiogenesis in vivo by directly controlling the transcription of vascular endothelial growth factor A (VEGFA). The results demonstrated that KLF5 deficiency could alter the paracrine properties of BC and affect interactions between BC and human umbilical vein endothelial cells (HUVECs). KLF5 may regulate VEGFA, a KLF5 target that mediated KLF5’s role in angiogenesis, expression, and tumor angiogenesis in vivo [96]. In conclusion, KLF5 stimulates the transcription of VEGFA, which directly influences BC angiogenesis. Therefore, KLF5 may be a potential therapeutic target in BC.

### Macrophage migration inhibitory factor (MIF)

The cytokine macrophage migration inhibitor factor (MIF), is an immunomodulator produced by glucocorticoids. It has been widely shown that MIF has a role in encouraging the invasion and metastasis of cancerous tumor cells [96].

Studies have revealed that the expression of MIF mRNA in bladder transitional cell carcinoma is higher than that in surrounding and healthy tissues. MIF may play a significant role in the development of lymphatic metastasis, the construction of microvascular structures, and the expression of VEGF in the tumor tissue. Expression of MIF is also higher in the index groups of high-stage and lymph node metastasis, which indicate high malignancy and poor prognosis, which is comparable with the findings in colon cancer [97]. These findings imply that MIF may facilitate tumor invasion and metastasis and contribute significantly to the occurrence and progression of BC. According to another study, bladder transition cell carcinoma (BTCC) may develop, invade, metastasize, and recur due to the high expression of MIF and VEGF and their synergistic effect. For molecular targeted therapy of BC, further study into its mechanism of action may represent a new turning point.

### FGFR

The receptor tyrosine kinase (RTK) subfamily FGFR has a high degree of sequence homology. There are five receptors in the human FGFR family (FGFR1-5). The signal axis of Fgfr1-4, which consists of an extracellular region, a hydrophobic transmembrane region, and an intracellular tyrosine kinase domain, is implicated in physiological activities like cell division, proliferation, tissue healing, and angiogenesis [98].

FGFR now contributes to the treatment of BC mostly because of its role in predicting the prognosis of BC and contributing in a present role to targeted therapy for BC. The FGFR3 gene was originally found in cancer in 1999 by Cappellen et al. [99], specifically in BC. The amounts of mutant FGFR3 mRNA in BC tissue samples were higher than those in normal urothelial specimens. In addition to inhibiting ligand binding, the specific FGFR3 monoclonal antibody (R3mAb) created by Qing et al. also blocks the dimerization of wild-type and different mutant FGFR3, which prevents the growth of BC RT112 and RT4 cell lines and prevents phosphorylation of FGFR3 and p42/44 MAPK [100]. In 75% of non-muscle invasive bladder tumors, FGFR3 activating mutations were found, and in 50% of muscle invasive high-grade bladder cancers, FGFR3 overexpression was found. The ability of BC cell lines to proliferate, form colonies, and invade can be inhibited by the knockdown of the mutant FGFR3 gene. Mutant FGFR3's oncogenic status was also supported by in vivo tests. The results of the BLC2001 clinical trial led to FDA approval of erdafitinib for patients with locally advanced and unresectable or metastatic BC who carry FGFR alterations and progress after platinum-based chemotherapy [101]. In the next few years, we will await the results of trials with other FGFR inhibitors, which may create an interesting, albeit complex, therapeutic opportunity. In addition, future studies will help to clarify the role of FGFR inhibitors in adjuvant or neoadjuvant therapy [102].

## The anoxic microenvironment in BC

**Fig. 4:**
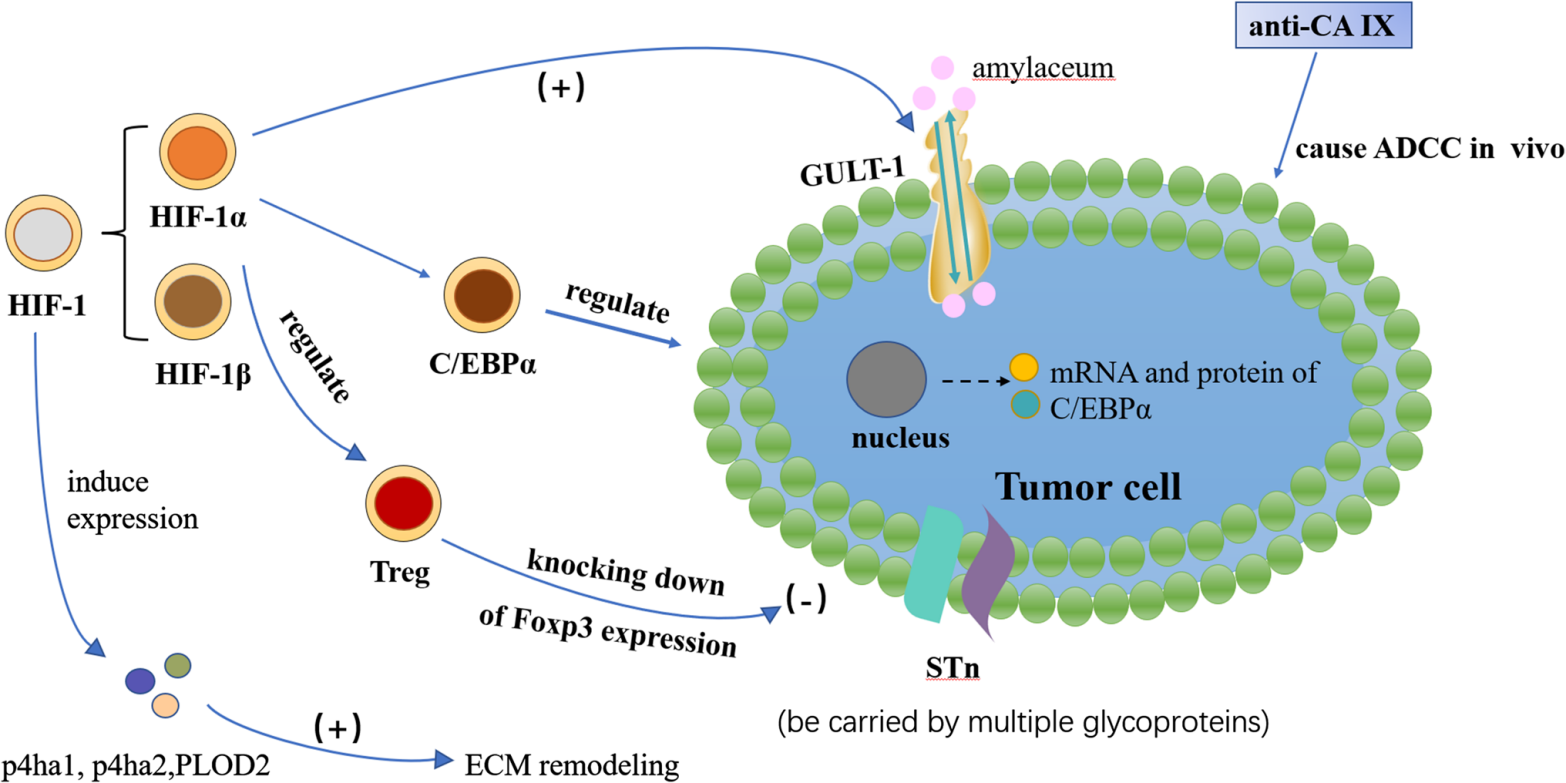
Anoxic microenvironment in bladder cancer. GLUT-1 is a kind of transmembrane protein distributed on the cell membrane and is a carrier for glucose to enter the cell. HIF-1 is a kind of transcription factor expressed by HIF-1 α and HIF-1 β under hypoxic stress. HIF-1 alpha upgrades many gene products, including the glucose transporter protein 1 (Glut-1). HIF-1 can promote ECM remodeling under hypoxia by inducing the expression of p4ha1, p4ha2, and PLOD2 in fibroblasts. The expression and localization of C/EBP alpha were regulated by hypoxia through a HIF-1 alpha-dependent mechanism. Carbonic anhydrase (CA) can be detected in almost all tissues of mammals and CAIX was highly expressed in bladder cancer tissues. CAIX monoclonal antibody (referred to as anti-CA IX) can cause antibody-dependent cell-mediated cytotoxicity (ADCC) in vivo. The forkhead box P3 (Foxp3) is an X-linked transcription factor. Foxp3 expression in Treg cells can be regulated through proteasomal degradation mediated by HIF-1α under hypoxic and normoxic condition. Knocking down of Foxp3 expression may block in vivo tumor growth

A supply of oxygen is necessary for the occurrence and development of tumors. Rapid tumor cell proliferation can result in the formation of an anoxic microenvironment, and while anoxia can inhibit tumor cell growth, tumor cells in anoxic microenvironments can choose more adaptable mechanisms, such as switching to a glycolytic metabolic mode, which results in unrestricted replicative potential and genetic instability (Fig. 4). As a result, this microenvironment promotes the progression and metastasis of the tumor.

### Hypoxia-inducible factor 1 (HIF-1)

HIF-1 is a transcriptional activator produced by cells under hypoxic stress and is divided into HIF-1α and HIF-1β. Many gene products, including glucose transporter protein 1, are upregulated by HIF-1α. Immunohistochemical studies utilizing a monoclonal antibody specific for HIF-1α have shown that the most common types of human cancer, including bladder cancer, exhibit upregulation of HIF-1α. A positive correlation was observed between the degree of tumor malignancy and the expression level of this factor [103]. HIF-1 can promote extracellular matrix remodeling under hypoxia by increasing the expression of p4ha1, p4ha2, and PLOD2 in fibroblasts, subsequently impacting cell shape, adhesion, and directional migration, creating a physical pathway for tumor invasion [104].

A study demonstrated that hypoxia in bladder cancer cells regulated the expression and localization of C/EBPα through a HIF-1α-dependent mechanism, which may be important for bladder cancer cell differentiation and proliferation [105]. The results showed that, whereas the mRNA levels of HIF-1 alpha were unaffected by hypoxic circumstances, the protein expression levels were noticeably increased. However, the localization of C/EBP alpha, which was noticeably reduced in the nucleus under hypoxic conditions, was significantly affected by the mRNA and protein levels of C/EBP alpha. In hypoxic BC cells, C/EBP alpha was a downstream effector regulated by HIF-1 alpha, and this regulatory pathway may be a viable therapeutic target in the treatment of BC.

### GLUT-1

A type of transmembrane protein found throughout the cell membrane is GLUT-1. In addition to being a target gene for HIF-1, it transports glucose into the cell. Numerous studies demonstrate that GLUT-1 expression and metabolic rate are higher in malignant tumors than in normal tissues, including head and neck tumors, lung cancer, and breast cancer. Since glucose is the primary source of energy for tumors, the process by which glucose is transported through membranes is crucial. A crucial role in the development of tumors is the bidirectional transport of the glucose transporter Glut-1 on the cell membrane [106]. A poor prognosis and a low survival rate are associated with Glut-1 expression in human BC. While urothelial BC frequently exhibits positive staining, nonneoplastic bladder urothelium typically exhibits GLUT-1 negativity. Additionally, several studies have found that GLUT-1 positive is a poor predictive factor [107].

### Carbonic anhydrase (CA)

Almost all mammalian tissues contain carbonic anhydrase (CA). According to recent research, the majority of tumors express carbonic anhydrase, and the isoenzyme expression varies depending on the kind of tumor tissue [108]. Most malignancies’ invasion and metastasis are directly correlated with this enzyme's expression [109]. The expression of carbonic anhydrase also had an impact on the prognosis and overall survival rate of cancer patients [110]. Regulating related carbonic anhydrase expression in cancer tumors may inhibit cancer cells from metastasis and improve the surgical prognosis for cancer patients [111, 112].

According to research by Klatte et al. [113], CA IX was nearly completely absent from normal bladder tissues but was substantially expressed in BC tissues and significantly correlated with patients' poor prognoses for survival. Different types of BC had different CA IX expression levels: non-invasive tumors expressed CA IX significantly more than invasive tumors, poorly differentiated tumors expressed CA IX more than well-differentiated tumors, and primary tumors of cancer tissues expressed CA IX significantly less than metastatic tumors of the same tumor tissues. There are currently a few targeted treatments based on monoclonal antibodies against CA IX. CA IX monoclonal antibody (also known as anti-CA IX) is capable of causing antibody-dependent cell-mediated cytotoxicity (ADCC) in vivo, making it useful for tumor immunotherapy.

### Sialyl-Tn (sTn)

Sialyl-Tn (sTn) is a truncated O-glycan associated with a poor prognosis and unfavorable outcome in cancer patients. Numerous glycoproteins are thought to transport sTn, which has the variety to affect protein function and play a role in tumor metastasis [114]. Bladder tumors can overexpress the cancer-associated carbohydrate antigen sTn, mimicking other advanced stage solid tumors and being extremely hypoxic [115, 116]. It is expressed in non-proliferative regions of tumors [117] rather than in healthy urothelium. The molecular foundation for future therapeutic invasion may be created by using a monoclonal antibody to target the sTn antigen and give the required tools to prevent invasion [118].

### Forkhead box P3 (Foxp3)

An X-linked transcription factor called forkhead box P3 (Foxp3) is necessary for the induction of immunosuppressive activities in regulatory T lymphocytes [119]. Foxp3 expression in Treg cells can be regulated through proteasomal degradation mediated by HIF-1α under hypoxic and normoxic condition [120]. Most studies viewed Foxp3 expression as playing an unfavorable role that is associated with lymph node or visceral metastases. Foxp3 expression knockdown may stop in vivo tumor growth, according to experiments [121]. A new possible target for establishing a new therapeutic approach for BC is provided by Foxp3.

## Exosomes in BC

Exosomes are extracellular vesicles that are released into the body when they fuse with the cell membrane. They are small, protein- and lipid-rich carriers that also contain RNA and other substances, with a diameter of about < 150 nm. Exosomes can trigger signal transduction through contacts between receptors and ligands because they carry surface molecules; alternatively, they can be internalized by endocytosis and/or phagocytosis and can even fuse with target cell membranes to transport their contents into the cytoplasm. As a result, exosomes from donor cells have the power to change the physiological conditions of recipient cells. Exosomes from non-tumor cells in particular have the ability to promote the adaptation of disseminated cancer cells to the external environment, demonstrating the occurrence of dynamic changes in the metastatic microenvironment [122].

**Fig. 5:**
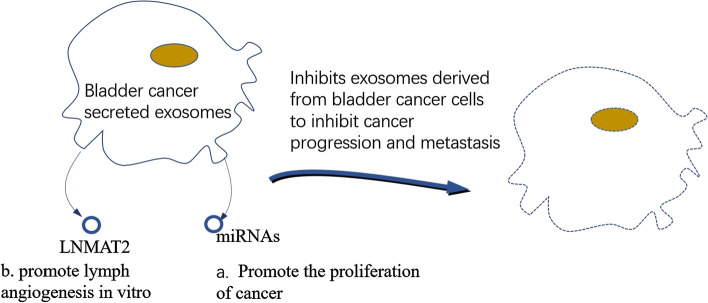
Mechanisms associated with exosomes secreted by bladder cancer. MiRNA and LNMAT2 in exosomes have the effect of promoting lymph angiogenesis and cancer cell metastasis, we can inhibit the progression and metastasis of bladder cancer by inhibiting the activity of exosomes

Urine cytology and cystoscopy are still the two main clinical methods for identifying and monitoring the progression of BC. Due to the intrusive nature of cystoscopy, there is an urgent need to find useful molecular markers that can be employed in its place. Many exosome components, including some microRNA species that are contained within the exosome lumen, are simply not present as free soluble molecules in bodily fluids. Therefore, the ability to separate exosomes from urine, plasma, saliva, or other physiological sources offers tremendous potential for acquiring unique and complex sets of biomarkers in a non-invasive manner. As such, exosome analysis may be useful for disease monitoring and diagnosis in BC [123]. Some of the mechanisms associated to the exosomes secreted by BC are shown in Fig. 5.

Exosomes are absorbed by cells, where they play a variety of functions through miRNAs in exosomes. As a result, understanding how exosomal miRNAs contribute to cancer and development is important for developing more effective treatments. MiRNAs play a role in numerous physiological processes, including cell division, proliferation, and apoptosis. They control the expression of target genes at the post-transcriptional level. Relevant studies have found that miRNAs play an important role in the occurrence and development of cancer, including tumor growth, invasion, and metastasis [124]. Exosomes that contain miRNA can shed from parental cells and travel to other parts of the body, suggesting that they can be used as excellent non-invasive biomarkers for cancer [125]. By targeting proteins or signaling pathways involved in the cell cycle, exosomal miRNAs secreted by tumors target to promote the proliferation of cancer cells. They also participate a role in the control of apoptotic signaling pathways in cancer cells at the same time.

Exosomal LNMAT2, which is secreted by BC cells, enhanced lymph angiogenesis in culture. The rate-determining stage for LN metastasis in BC is lymph angiogenesis [126]. Analysis was done on the tube formation and migration in HLECs that were treated with exosomes secreted by BC cells. Compared to the control, the exosomes secreted by BC cells significantly aided HLEC tube development and migration. Additionally, HLEC tube formation and migration were highly promoted by the exosomes secreted by LNMAT2-overexpressing UM-UC-3 cells ((UM-UC-3-EXO_LNMAT2_). The ability to promote HLEC tube formation and migration was lost by exosomes secreted by 5637 cells (5637-EXO_si-LNMAT2_) that were LNMAT2-silenced. These results suggest that exosomal LNMAT2 participates in lymph angiogenesis in cultured lymphocytes. By directly interacting with heterogeneous nuclear ribonucleoprotein A2B1(hnRNPA2B1), LNMAT2 was loaded to BC cell-secreted exosomes in a specific manner. Exosomal LNMAT2 was then internalized by HLECs, and by enlisting hnRNPA2B1 and raising the H3K4 trimethylation level in the PROX1 promoter, epigenetically increased prospero homeobox 1 (PROX1) expression was then induced, leading to lymph angiogenesis and lymphatic metastasis [127].

## Important signaling pathways in BC

The term "signaling pathway" refers to a series of enzymatic reaction pathways that allow extracellular molecular signals to transfer cells and function via the cell membrane. Hormones, growth factors, cytokines, neurotransmitters, and other small molecule substances are some examples of these extracellular molecular signals. The metastasis of BC is correlated with signaling pathways, and some genes may speed up BC progression via these pathways. Various signaling pathways have been well defined.

### TGF-β pathway

Cell proliferation, differentiation, adhesion, senescence, and apoptosis are all influenced by a variety of distinct effects of the Transforming Growth Factor β (TGF-β) signaling pathway. TGF-β is widely produced by a variety of immune and non-immune cells, and it controls cellular function in both an autocrine and paracrine manner. It plays crucial roles in biological processes such as tumor progression, immune response, and embryonic development. Only a few cell signalings in mammals, like the TGF-β signaling pathway, can contribute as many pleiotropic activities [128].

In early-stage tumors, the TGFβ pathway acts as a tumor suppressor by inducing apoptosis and preventing the proliferation of carcinoma cells. On the other hand, it exerts pro-tumor effects in the late stages by influencing immune evasion, neo-angiogenesis, genomic instability, epithelial-mesenchymal transition (EMT), cell motility, and metastasis. As an immunosuppressive cytokine, TGF-β exerts broad-reaching inhibitory effects on the immune response through a variety of mechanisms. It is widely acknowledged that TGF-β-induced EMT plays a key role in carcinoma invasion and metastasis [129]. In addition, prior research demonstrated the critical role played by TGF-β-induced signaling via the PI3K-PKBmammalian target of rapamycin (mTOR) pathway in the progression of EMT, suggesting that mTOR may be a good candidate for use as a target in the fight against cancer metastasis [130]. Interesting research has revealed that circRIP2 speeds up BC progression by acting on the miR-1305/TGF-β2/smad3 pathway. By generating EMT and activating the miR-1305/TGF-β2/smad3 pathway, effective circRIP2 activation hastens the progression of BC. According to the research, circRIP2 might be a promising biomarker and therapeutic target for BC patients [131].

In addition, the TGF-β pathway can also modulate the metabolic reprogramming of cancer cells and their microenvironment, affecting the availability and utilization of nutrients, oxygen, and energy [132].

### IL-1 pathway

Long recognized for its pleiotropic effects on inflammation, interleukin 1 (IL-1) also plays a complex, and occasionally conflicting, role in several stages of cancer development. IL-1β is a key cytokine that promotes inflammation and is mostly expressed by innate immune cells. However, different cell types both in healthy and unhealthy situations express IL-1α. The primary receptor for both ligands is IL-1R1, which is expressed by many different cell types, such as innate and adaptive immune cell types, epithelial cells, endothelial cells, adipocytes, chondrocytes, fibroblasts, etc. The complicated positive and negative regulatory mechanisms of IL-1 and IL-1R1 receptor contact result in a set of common signaling pathways, primarily the NF-kB and MAP kinase pathways. The various cell types that express IL-1R1 control the function of IL-1 signaling at various stages of cancer, which in some cases results in divergent roles in tumor development [133].

Studies show an inverse role between IL1RA expression and tumor cell invasiveness and migration, suggesting that IL1RA contributes to bladder carcinogenesis, however the precise mechanisms by which IL1RA affects tumor cell migration and invasion are still unknown [134].

### IL-8 pathway

IL-8 is a multipurpose cytokine that was first discovered as a protein that attracts neutrophils. Numerous tumor cells, lymphocytes, neutrophils, macrophages, and other cell types can all secrete IL-8 [135]. The in vitro co-culture assay also demonstrated that B cells could be recruited more easily towards BC cells compared to normal bladder cells, and it was found that BC tissues could attract more B cells than the surrounding normal bladder tissues in human clinical BC samples. By increasing IL-8/androgen receptor (AR) signals in BC cells, recruited B cells were able to promote the expression of metastasis genes including MMP1 and MMP13. A partial reversal of the filtering B cells' potential to increase BC cell invasion was achieved by blocking the IL-8/AR/MMPs signals with anti-IL-8 neutralizing antibodies, AR-siRNA, or MMPs inhibitors, respectively [136]. It has been suggested that IL-8, which controls tumorigenicity and metastasis in human BC [137], can be used as a biomarker for the detection of BC in the urine. Fascinatingly, IL-8 has also been linked to increased AR transcriptional activity and may promote prostate cancer progression through an androgen-free pathway. According to studies, IL-8 could be the AR upstream signaling to modify AR in BC cells, and B cell recruitment to BC cells could further increase IL-8 expression [138]. It is unknown whether the mechanism can be applied to BC.

### PI3K/AKT/mTOR pathway

One of the most extensively researched therapeutic targets for treating cancer is the phosphoinositide 3-kinase (PI3K)/AKT/mammalian target of the rapamycin (mTOR) pathway. In cancer, the PI3K/AKT/mTOR signaling pathway experiences regular molecular changes and increases in activity. Molecules in this pathway are interesting targets for pharmacological intervention because of their role in the regulation of cell growth, survival, and metastasis. It has been shown in numerous unrelated studies that muscle-invasive or metastatic BCfrequently overactivated the PI3K signaling pathway. In 21–25% of muscle-invasive BC, PIK3CA mutations (which code for the p110α subunit of PI3K) are present [139]. The RTK/RAS/PI3K/AKT/mTOR pathway is changed in 72% of BLCA, according to statistics. Due to the high incidence of PI3K pathway signaling dysregulation and the availability of a number of small molecule inhibitors to block it, PI3K signaling has gained attention as a potential therapeutic target in BC.

The PI3K/AKT/mTOR pathway can affect the microenvironment of bladder cancer in several ways [140], such as stimulating the production of growth factors, cytokines, and chemokines that promote tumor growth, invasion, and angiogenesis, enhancing the expression of matrix metalloproteinases (MMPs) and integrins that degrade the extracellular matrix and facilitate tumor migration and adhesion and suppressing the anti-tumor immunity by inhibiting the activation and function of T cells, natural killer cells, and dendritic cells. It can also modulating the metabolic reprogramming of cancer cells and their microenvironment, affecting the availability and utilization of nutrients, oxygen, and energy [141].

### WNT/β pathway

Previous studies have shown that the WNT/β-catenin signaling pathway is closely related to the epithelial-mesenchymal transition and tumor progression. Furthermore, the translocation of the transcription factor β-catenin from the cytoplasm to the nucleus can activate its target genes, including MMP7 and c-Myc, which are beneficial for BC metastasis and tumor progression [142].

### Notch pathway

Notch pathway has been found associated with many types of malignancies including BC. The pathway's ability to be either tumor-suppressive or oncogenic depending on the type of tumor is one of its special characteristics. Notch signaling regulates the TME by acting on macrophages and MDSCs and by directly modulating the cytotoxic capacity of CD8 + T cells. How these mechanisms are directly regulated by the ligands expressed in the TME requires further investigation [143]. For instance, in T-ALL, B-cell chronic lymphocytic leukemia, or lung adenocarcinoma, NOTCH receptors present gain-of-function mutations that make the receptor constitutively active.

Surprisingly, research has revealed that loss-of-function mutations in NOTCH pathway components and NOTCH1 gene copy losses occur in up to 60% of bladder cancers [144]. This is supported by subsequent cancers that demonstrated the functional insufficiency of NOTCH1 and NOTCH2 mutations that were previously found in bladder cancers [145]. Relevantly, both studies found a connection between decreased Notch pathway activation, higher cancer aggressiveness, and shorter patient survival.

### Sex hormone receptor signaling pathway

Sex steroid hormone-mediated signaling plays a significant role in the development of bladder cancer. Sex hormone receptors, including estrogen receptors (ER) and androgen receptor (AR), show their functional activities in influencing BC progression.

#### AR

Androgens, such as dihydrotestosterone (DHT) and methyltrienolone (R1881), have vital impact on the cell proliferation, migration and invasion in BC [146–148]. AR targeted on some potential downstream molecules or pathways to cause a ripple in the progression of BC. For example, AR may active ATF2 [149], WNT/β pathway [150], CD24 [151] and vascular endothelia growth factor (VEGF) [152]. Actually, these molecules or pathways have been implicated in the apoptosis, invasion and metastasis of BC. The downstream molecules or pathways may be the molecular mechanisms for how AR promote the BC progression. Correspondingly, knockdown of AR or AR antagonists, such as enzalutamide, bicalutamide and flutamide show the inhibitory effects, which has been proved effective treatments of BC.

#### ERs

There are two types of nuclear ERs exist in humans: ERα and ERβ. These two receptors may differ in the biological function especially in BC. One of the most remarkable is that ERα has no prognostic significance [153, 154], while the expression of ERβ has been found up-regulated in high-grade BC and was associated with the metastasis and recurrence.

Moreover, ERα and ERβ play different role in the growth of BC. A research about the knockdown of ERα found that the low expression of ERα could induce the growth of BC cells [155], which suggested its inhibitory function. On the contrary, diatylpropionitrile, used to inhibit the expression of ERβ, has been proved effective inhibiting the migration and invasion of BC.

ERs function in BC could be explained by its molecular mechanisms. Researches have revealed that ERs could potentially modulate pathways such as AKT/ERK [156], E-cadherin/N-cadherin [157] and MCM2, all of which are known to involve the progression of BC [132].

## Treatment of BC

A summary of current treatments for BC is presented in Table 4. In this section we will focus on immunotherapy and Antibody therapy of TME-related.

**Table 4:** Treatment of bladder cancer. This table shows several common treatment options for 0

Serial number	Treatment of bladder cancer	Representative therapy	References
1	Surgical treatment	Transurethral resection of bladder tumor (TURBT)	[158]
		Radical cystectomy (RC)	[159]
		Bacillus Calmette-Guérin (BCG)	[160]
2	Chemotherapy	Gemcitabine with cisplatin	[218]
		Dose-dense methotrexate, vinblastine, doxorubicin, and cisplatin (ddMVAC)	[219]
3	Radiation therapy	External beam radiation therapy (EBRT)	[220]
4	Targeted therapies	Erdafitinib (FGFR3 inhibitor)	[102]
		Sacituzumab Govitecan	[221]
5	Immune checkpoint inhibitor	AbPD-1	[162]
		AbCTLA-4	[168]
6	Agonistic antibody therapy—targeting costimulatory molecules (experimental)	OX40	[178]
		CD40	[184]
7	Adoptive T cell therapy (ACT) (experimental)	CAR-T cell 1024 Therapy, TIL therapy	[173]
8	Anti-angiogenesis therapy	Cabozantinib	[185–190]
9	Antibody–drug conjugates (ADCs)	Antibody EV targeted nectin-4 and linked to vedotin	[198]
10	Gene therapy	Adstiladrin	[222]

### Immunotherapy

#### BCG

Bacillus Calmette-Guérin (BCG), a non-specific immune stimulant, was originally utilized by Morales to treat superficial BC in 1976. Although the exact mechanism is uncertain, intravesical BCG causes the release of interleukin-1, interleukin-2, and tumor necrosis factor along with a local host immune response directed towards tumor cells. For patients with Ta and T1 disease, intravesical BCG has been shown to slow tumor progression and recurrence rates and to bring about a full remission for patients with Tis. Intravesical BCG may have local (cystitis) and/or systemic side effects (fever, malaise, nausea) [160].

BCG needs to be administered more than once in order to cause a BCG reaction. The induction phase usually lasts six weekly instillations, some patients respond better to fewer installations while others need more. A maximum of two consecutive cycles of induction treatment are advised. A second induction cycle can be tried if the first cycle yields an inadequate response [161]. When the immunologic reaction has taken place, the induction phase is said to be finished. This is demonstrated by the fact that the patient exhibits symptoms of an irritable bladder, that there are white blood cells in the urine but no sign of an infection and that microscopic hematuria is present.

#### Immune checkpoint inhibitors (ICIs)

More than 75% of patients with non-muscle invasive cancer need localized conservative treatment, while the remaining 25% of patients receive radical cystectomy or radiotherapy. By blocking inhibitory receptors and ligands expressed on antigen-presenting cells, T lymphocytes, and tumor cells, immune checkpoint inhibitors offer a novel family of immunotherapy medications that restore natural antitumoral immune function [162]. Immune checkpoint inhibitors are still being explored with the development of immunotherapeutic pathways and related markers.

Despite the considerable curative effect, clarifications of ICIs’ toxicity are still required. The clarifications will be presented in the next text.

*AbPD-1* The ligand (PD-L1), which is expressed on activated T cells, natural killer (NK) cells, APC, and tumor cells, interacts with the coinhibitory receptor (PD-1) to cause activation. PD-1 is a coinhibitory receptor that inhibits T cell function [163].

Anti-PD-1 antibody pembrolizumab has a variety of clinical uses [162]. Another anti-PD1/PDL1 antibody, atezolizumab, was authorized for the treatment of metastatic non-small cell lung cancer (NSCLC) and urothelial cancer in 2016. The ABACUS trial, a single-arm phase II study, examined the use of two cycles of atezolizumab (1200 mg per cycle) every three weeks in 95 MIBC patients who were ineligible to receive cisplatin prior to cystectomy. The patients have shown considerable pathological complete response (pCR) despite the lack of a meaningful correlation between PD-L1 expression and prognosis [164].

The toxicity of AbPD-1 should be highlighted. The most common all-grade adverse events were fatigue, pruritus and diarrhea. What’s more, immune-related adverse events happened, including hypothyroidism, colitis, hypophysitis and perhaps pneumonitis [165]. Compared with PD-L1 inhibitors, PD-1 inhibitors may lead to higher adverse events [166].

*AbCTLA-4* During the interaction between the TCR, CD28, and B7, the competitive receptor for CD28, CTLA-4, is increased on the surface of T cells. To offset the stimulatory signals of CD28/B7 and TCR/MHC-II, the binding of CTLA-4 to B7 elicits an inhibitory signal that dampens TCR signaling, which stops T cell proliferation and suppresses the production of IL-2. 158 Tumor cells are able to express CTLA-4 to trigger the transmission of an apoptotic signal to T cells, although the particular mechanisms and pathways involved still need to be completely understood [167]. Furthermore, PD-L1 could be stimulated by CTLA-4 tumor expression [168].

Ipilimumab, an anti-CTLA-4 antibody that is frequently used to treat melanoma, was the first checkpoint inhibitor to be applied in a preoperative setting for MIBC. In a study that was published in 2010, localized urothelial carcinoma patients with cT1-T2N0M0 were given two cycles of Ipilimumab (up to 10 mg/kg) before surgery [169]. The study showed promising early outcomes; positive urine cytology became negative, and lower-stage disease was seen on surgical specimens compared to pre-immunotherapy transurethral resection specimens. Only rash and diarrhea were reported as adverse effects. Ipilimumab is expected to be used to treat BC.

AbCTLA-4, especially the ipilimumab, were associated with the risk of fatal gastrointestinal toxicity [170]. Among all the fatal adverse events, diarrhoea, colitis and perforation are the most common ones. Ipilimumab may block CTLA-4, inhibiting the effect of regulatory T cells in the gastrointestinal tract [171].

#### Adoptive T cell therapy (ACT)

For the past 10 years, immunotherapy has been utilized to treat BC [172]. CAR-T cell therapy and TIL therapy are the two most common adoptive T cell therapies. Adoptive immunotherapy is thought to be typified by CAR-T cells [172]. The host immune response will be able to recognize tumor cells thanks to CAR-T therapy, which uses genetic engineering to generate specific T-cell receptors [173]. It calls for the removal of immune T cells from patients and the genetic alteration of these cells in a lab setting so that they can be loaded with "chimeric antigen receptors" (CAR) that can recognize the surface antigens of cancer cells. Because urologic neoplasms grow rather slowly in systemic tumors, there is sufficient time to employ CAR-T cells during this process. Multiple CAR-T therapies with various targets can be carried out since urologic neoplasms have unique pathogenesis characteristics. These altered cells were then significantly multiplied in the lab and reinjected back into the patient. CAR-T cells still have a poor ability to invade and stay in urologic neoplasms due to the immunosuppressive microenvironment and physical obstacles present in tumor tissue. The use of CAR-T in urologic neoplasms also raises important questions about targeting extra tumoral cytotoxicity. As a result, pertinent studies must improve the target selection, and CAR-T cells may be better able to eradicate urologic neoplasms through gene-editing cytokines, combined molecular targeting drugs, and chemotherapy [174]. Additionally, findings demonstrate that decitabine combination with CART cell therapy is a promising new therapy that can improve BC-specific tumor killing [174].

Tumor Infiltrating Lymphocytes (TIL) are a kind of heterogeneous lymphocytes that include T cells and NK cells in tumor stroma. These cells are able to recognize, fend off, and fight tumors most effectively by directing the immune system to penetrate deeply into the tumor tissue after identifying cancer cells in the body. When it gets within the tumor, it releases cytotoxins that directly kill the tumor cells [175]. Additionally, there are numerous ways that TIL therapy can be given; for example, systemic administration can treat patients who have metastatic BC or who have had a cystectomy, while intravesical delivery can treat patients who have NMIBC as a treatment-sparing option. TIL might be administered to MIBC patients in an adjuvant or neoadjuvant context. The combination of systemic immune checkpoint inhibitors and systemic TIL therapy has been demonstrated to improve the efficacy of ACT in patients with metastatic melanoma. We predict that intravesical TIL administration would eliminate the need for systemic IL-2 and non-myeloablative chemotherapy (NMAC). In addition, intravesical TIL therapy has the ability to be administered repeatedly and might be used in conjunction with intravesical IL-2 without the severe side effects associated with systemic IL-2 [175].

ACT has shown accredited effect as a viable treatment for solid tumors. There are a number of promising cell-based treatments that have been created for BC. A lot of pro clinical models have been tested to ensure the reliability of ACT.

### Antibody therapy for TME molecules

Therapies below similarly target at specific molecules in TME. Using diverse antibodies, these therapies may guide a new direction of BC treatments.

#### Agonistic antibody therapy—targeting costimulatory molecules

*OX40* OX40 is a potent co-stimulatory receptor of immune responses in various cancers and has been used as a target for the generation of agonists of its function, a member of the tumor necrosis factor receptor (TNFR) superfamily, which is involved in activated CD4, CD8 T cells and It is expressed on several other lymphocytes and non-lymphocytes and is involved in the activation, proliferation, and migration of T cells, as well as the formation of germinal centers and the differentiation and maturation of dendritic cells [176, 177]. Through agonists, OX40 and its ligand OX40L can take part in immune modulation and play a critical role in modulating tumor immune responses and the development of autoimmune disorders [178]. The study demonstrated that bladder cancer can be successfully treated with CpG and agonistic anti-OX40 treatment. This research serves as a foundation for future studies using TLR agonists and antagonists, which can target the immune response to the cellular pathway [179].

*CD40* The tumor necrosis factor receptor superfamily includes the transmembrane glycoprotein CD40. The studies have demonstrated that the CD40 molecule was present on the surface of antigen presentation cells (APC) [180], gastric cancer [181], normal BC [182], colon cancer [183], and various solid tumors and hematological tumor cells. In the process of carcinogenesis and tumor development, CD40 molecules are expressed differently. According to the study, dendritic cells (DCs) in the microenvironment of bladder tumors in orthotopic BC animal models had high expression levels of the immune-stimulatory receptor CD40. Moreover, they show that local CD40 agonism in mice with orthotopic BC caused by the intravesical administration of anti-CD40 agonist antibodies triggers powerful antitumor immunity and has pharmacodynamic effects on the bladder tumor microenvironment [184].

#### Anti-angiogenesis therapy

It has been established that an angiogenesis-promoting microenvironment is important for BC growth and metastasis. The prognosis of diseases with metastatic spread is associated with the biomarkers. Vascular endothelial growth factor receptor 1 (VEGFR1) and VEGFR2 and their ligands (VEGF-A through VEGF-D) promote angiogenesis and play a crucial role in the pathogenesis and progression of BC; as a target, they can be targets for the anti-angiogenesis therapy.

Some VEGF/VEGFR inhibitors have been tested as single agents and have demonstrated limited anticancer activity [185–190]. Only when used in conjunction with other drugs, VEGF/VEGFR inhibitors show effect. Ramucirumab, a monoclonal antibody that targets VEGFR-2 [191], has been tested on several tumor types and exhibited a higher objective response rate when combined with docetaxel than all other drugs combined. What is negligible is that Ramucirumab nonetheless demonstrated a high rate of severe adverse events [192].

Due to their potential for immunomodulation, ICIs and VEGF/ VEGFR inhibitors are another prospective pairing [193]. A VEGFR2/c-MET/RET tyrosine kinase inhibitor called cabotinib has demonstrated a higher objective response rate in the study [194].

#### Antibody–drug conjugates

Antibody–drug conjugates (ADCs) are immune conjugates that attach to monoclonal antibodies and cytotoxic medications through chemical connectors [195–197]. ADCs are made up of three parts: a chemotherapeutic drug, a protease-cleavable linker, and a monoclonal antibody against a target that is often expressed in cancer cells [198].

Highly expressed tumor proteins are used by ADCs as drug delivery targets [199]. On the tumor cell surface, BC has a number of highly expressed proteins like Her-2, Trop-2, and others. Hence it makes sense to use ADCs in therapy. Furthermore, the cytotoxic substance can only be released inside cells after internalization of ADCs and subsequent lysosomal linker cleavage. As a result, the formulation might deliver significant dosages of chemotherapy in a focused manner. In addition, by triggering the complement system and immune effector cells at the tumor site, not only are the targeted cells affected, but also nearby tumor cells and the stromal tissues that surround them [195].

Monoclonal antibody EV, also known as ASG-22ME, targets nectin-4 and is linked to vedotin. EV has demonstrated an impressive objective response rate during the clinical study despite significant adverse effects [200, 201]. Sacituzumab govitecan, which targets Trop-2, is positive in the treatment of locally advanced muscle-invasive bladder cancer [202]. The Her-2-targeting ADC DS-8201a, which is associated with the new topoisomerase I inhibitor DXd, is a different ADC that is still in the early stages of clinical development.

In summary, antibody–drug conjugates have demonstrated impressive effects in the target therapy for BC. ADCs is a new approach to treating BC that is more effective and targeted.

## Conclusion and prospects

We examined the key elements of the immune microenvironment surrounding BC tumors as well as its key stromal cells in this systematic study. The hypoxic and angiogenesis-promoting microenvironments were also examined. In understanding to better understand BC and effectively treat disease, we have included provided a summary of several important molecular pathways. Several selected molecules and pathways have been highlighted, which provided new ideas for the treatment of BC. Numerous previous studies and Meta-analyses have clarified that therapies targeting at specific molecules would be a new trend of the researches on BC.

The understanding of the molecular biology and genetics of BC has been improved. Therefore, the way BC be diagnosed and treated is in progress. In the future, it is hoped that the complete picture of the tumor microenvironment will be defined, and the combination of immunotherapy with surgery, radiation, chemotherapy, and targeted therapies will finally become a reality to treat BC. This will happen as we continue to study the interactions between tumor cells and components of the tumor microenvironment.

However, this review still has limitations. First, clarification on TME is not clear, despite several microenvironments put forward and many molecules presented. Second, we limited the discussion to the most common types of BC. Although we treat different types of BC similarly, detailed pathways which drive distinct BC should be extensively discussed. With the rapidly changing of the therapy prosect of BC, our review may be lack of future options.

## Data Availability

The datasets used or analyzed during the current study are available from the corresponding author on reasonable request.
